# Australian sport and physical activity behaviours pre, during and post-COVID-19

**DOI:** 10.1186/s12889-024-18245-y

**Published:** 2024-03-18

**Authors:** Rochelle Eime, Jack Harvey, Melanie Charity

**Affiliations:** Physical Activity and Sport Insights, Research and Innovation, Federation University, Ballarat, Australia

**Keywords:** COVID-19, Sport, Patterns, Community

## Abstract

**Background:**

Globally, COVID-19 and associated restrictions impacted negatively on recreational physical activity (RPA). Participation in community sport was significantly impacted with cancelled training and competitions. Whilst team and club-based sport participation declined during COVID-19 restrictions, participation in some physical activities actually increased, particularly individual and online activities and outdoor activities not requiring facilities.

**Aim:**

The aim of this study is to investigate changes in the patterns of participation in club-based sport, informal sport and other RPA in Australia from pre, during and post-COVID-19 restrictions. Further, these participation patterns are broken down by gender, age and region of residence.

**Methods:**

Two longitudinal waves of an online survey were conducted in mid-2020 and mid-2021. The first wave also captured retrospective pre-COVID19 (2019) data. Two sections of the survey dealt respectively with two ‘settings’ of RPA: organised club sport, and less structured sport and recreational physical activity (designated ‘other RPA’). For each year 2019–2021 each individual was categorized as participating (Yes/No) in each of club sport and other RPA. For each setting, the proportions of each pattern of participation were tabulated, and the results for the demographic cohorts were compared.

**Results:**

A total of 1,138 Australians aged 13 years and above completed both waves of the survey. Overall, there were considerable differences between the patterns of club sport and other RPA. Most individuals who participated in other RPA (69%) were able to and did participate continuously throughout the COVID-19 pandemic. However, and not surprisingly, the club-sport participants were forced to drop out in 2020 during COVID-19 restrictions, and less than half reported returning to play post-COVID-19 restrictions. Less than a quarter of sports club participants were able to continue to play throughout COVID-19 and beyond. Significantly more males returned to playing sport 51% than females 44%.

**Conclusion:**

Participation in community club-based sport has been significantly negatively impacted by COVID-19, more so than participation in some other recreational sport and physical activities. Further, fewer females than males returned to playing community sport, and priority and specific attention should be given to understanding why women and girls have not returned to playing community club-based sport.

## Background

### Impact of COVID on sport and physical activity trends

Globally, the COVID-19 pandemic and associated restrictions impacted negatively on physical activity, with decreased participation consistently reported [1, 2]. Effects included decrease in both daily steps and daily moderate-to-vigorous physical activity, and increased time spent in sedentary behaviour [3, 4]. Community sport was also impacted heavily, with individuals no longer able to play, as competitions and trainings were cancelled due to COVID-19 restrictions [5–8].

An Australian study reported that participation in community sport declined by 27% in 2020 compared to 2019 and that the largest decline was for those aged 4–9 years [9]. Across age groups, 4 year olds decreased participation by 69%, 5–9 year olds by 38% and 10–14 year olds by 18% [9]. The decline in participation was greater for females than males [9] which is similar to other studies on leisure-time physical activity which reported greater declines in participation during COVID-19 restrictions for females [10].

During the first COVID-19 restrictions and lockdowns in Australia, the greatest decline in sport and physical activity participation was participation in team sports such as bowls, cricket and netball [6, 11]. However, participation in some sports and physical activities, particularly individual-based activities actually increased. This included increased participation in running/jogging, walking, yoga, bushwalking and cycling [6]. Similarly, other studies internationally also reported increased participation in individual activities, and specifically outdoor activities, when indoor activities were ceased [12].

With the absence of organised community club-based sport during COVID-19 restrictions, many individuals transitioned to virtual and home-based sport and physical activity and programs [5, 13]. Further, modern technology allowed many people to stay connected and be active together online, and allowed coaches and instructors to keep in touch with their players and clients [14]. Whilst many turned to online fitness programs and home workouts, many lost the motivation to be active [5, 15] and this led many individuals to become more sedentary [2, 5].

### Impact of COVID-19 sport and physical activity restrictions on health outcomes

It is important to understand how individuals, families and communities have rebounded now that COVID-19 restrictions on sport and physical activity have been lifted, because of the heavy impact the restrictions had on individuals’ health and wellbeing. The absence of community sport for Australian youth during COVID-19 restrictions impacted female physical and mental health significantly more than males [16]. The opposite was reported for adult and older adult respondents to the same survey, with men demonstrating significantly worse general, physical and mental health and lower life satisfaction than women [11]. For this cohort, the absence of playing competitive sport and training with friends, teams and within clubs more heavily impacted males and younger adults [11]. Amongst the older adults aged 60 and above, those who participated in both club-based sport and informal activities had significantly better general, physical health and resilience than those who participated solely in a single setting [17]. Further, those participating in both team and individual activities reported better general wellbeing [17].

### Return to sport and physical activity post-COVID-19 restrictions

Whilst there is an abundance of research on the impact of COVID-19 restriction on changes to physical activity behaviour during COVID-19, and some specifically on participation in sport, there is limited published to date on the patterns of participation in sport and physical activity post-COVID-19 restrictions. Not surprisingly, there are reports that post-COVID-19 restrictions, for some individuals, particularly among children, their sedentary behaviours during restrictions have become habitual [18]. Others have reported that not all participants have been returning to playing sport [15, 19]. It is consistently reported that the negative economic impact of COVID-19 has been a barrier to many returning to playing sport [14, 15, 19]. In Australia, overall trends in terms of the total number of individuals playing sport is similar in 2021 compared to pre-COVID [9]. However, females have been much less likely to return to playing sport than the males, with male participation actually higher post-COVID-19 (2021) compared to pre-COVID-19 (2019) [9].

The aim of this study is to investigate changes in the patterns of participation in club-based sport and informal sport and other recreational physical activity in Australia from pre, during and post-COVID-19 restrictions. Further these participation patterns are broken down by gender, age and region of residence.

## Methods

### Survey sample

This study is part of a broader program of research in Australia which involves the longitudinal measurement of sport and physical activity profiles and physical, mental and social health and wellbeing outcomes that are the result of this participation. This study was conducted in two waves of online surveying during the COVID-19 period (2020 and 2021). The first wave of data collection included retrospective (baseline) data pertaining to pre-COVID-19 participation rates in 2019.

Full details of the program of research can be found elsewhere [6, 11]. Briefly, recruitment to the survey was primarily facilitated by several national and state sporting organizations. The target population was people aged 13 years or older who were registered in the 2019 and/or 2020 playing seasons to participate in one or more sports. The sport organizations that sent out the survey invitation to their registered participants represent major sports in Victoria and Australia [20, 21].

This approach was supplemented by recruitment through social media, which resulted in an additional smaller sample of participants in only informal sport or other physical activity settings [22].

All participants in the first survey were invited to participate in a similar follow-up survey conducted one year later, in May-June 2022, and just over 20% did so. The present study is based on this group of respondents to both surveys.

Ethics approval was obtained from Victoria University (project number HRE20-049) human research ethics committee. Informed consent to participate was obtained by participants and in the case of adolescents, a parent/caregiver provided consent.

### Survey instrument

The first wave, or baseline, of the longitudinal survey included among other themes, questions about:

- Demographic characteristics– gender, age, and residential postcode.
- Types of sports and other recreational physical activities participated in.
- Frequency and duration of participation, at the time of the survey (May-June 2020) and during the previous year (2019).

The second wave survey included similar questions about types, frequency and duration of participation in May-June 2021.

### Analysis

Date of birth was used to determine age in years at the time the first survey was completed.Residential postcode correspondence Table [23] were used to assign each postcode to one of two broad geographical zones or regions: Metropolitan, comprising the capital cities of the Australian states; and Non-metropolitan, comprising regional cities, towns and rural areas.

Regarding recreational physical activity (RPA), two separate sections of the survey dealt respectively with two ‘settings’: organised club sport involving membership and registration (designated ‘Club sport’), and less structured sport and recreational physical activity (designated ‘Other RPA’). In each section, a list of the most common activities was presented– 16 for club sport and 26 for other RPA (which also included 12 of the 16 club sports). Respondents indicated the activities in which they participated, with provision for adding other activities that were not listed.

In the present study, each respondent was assigned, for each of the three years (2019, 2020, 2021) three dichotomous participation indicators (1 = participated, 0 = did not participate), based respectively on participation in each of the two types of RPA (‘Club sport’ and ‘Other RPA’) and participation in ‘Any RPA’ (i.e. Club sport, Other RPA or both). This set of nine indicators (three indicators for each of the three years) provides a ‘broad brush’ snapshot of the overall impact of the imposition of COVID-19 restrictions in 2020 and their subsequent easing in 2021 on levels of engagement in RPA generally, and more specifically, in club sport and in other forms of RPA.

For each of the three types/groupings of activity, the three annual dichotomous indicators together define a pattern or sequence of participation over the 3-year period. There are eight (2 × 2 × 2) possible sequences, of which four involve no participation in 2019. These four patterns were driven by various individual factors rather than COVID-19 restrictions, and each had low counts in some demographic categories, and so for purposes of analysis they were combined, reducing the number of patterns to five.

The participation patterns of all respondents were tabulated, and the patterns regarding club sport and other RPA were compared using a McNemar-Bowker test for multiple correlated proportions. Participation patterns of groups based in turn on gender, age and region of residence were tabulated and compared using chi-square tests of independence. Statistical significance was set at *p* =.05. Statistical analysis was conducted using SPSS Version 27.

## Results

Of 5,371 respondents who completed the sport and physical activity questions in the Wave 1 survey, 1,138 (21.2%) also completed the Wave 2 follow-up survey. The latter group provided the basis for this study. Collectively, these respondents reported 3,599 instances of participation (i.e., a person reporting that they participated in a particular activity), an average of 3.2 different activities per person, in a total of 65 sports and physical activities.

The great majority (1024 or 90.0%) were recruited by the sports organisations, and almost half of the 10% recruited through social media (48 or 4.2%) also had club sport affiliations. The small social media cohort was comprised entirely of adults (aged 18 years or more). It included higher proportions of women and participants in informal sport and other recreational physical activity than the sport organisation cohort.

**Table 1:** Patterns of participation in RPA^1^ 2019–2022: by type of RPA

Participation pattern	Participation sequence^2^	Any RPA		Club sport		Other RPA		p-value^3^
		Count	%	Count	%	Count	%	
Did not play in 2019	000 010 011 001	6	0.5	145	12.7	181	15.9	< 0.001
Dropped out 2020 no return 2021	100	24	2.1	136	**12.0**	17	1.5	
Dropped out 2020, returned 2021	101	156	13.7	544	**47.8**	39	3.4	
Dropped out 2021	110	50	4.4	46	4.0	117	**10.3**	
Continued 2019–2021	111	902	79.3	267	23.5	784	**68.9**	
N		1138	100.0	1138	100.0	1138	100.0	

^1^ Recreational Physical Activity

^2^ 0 = did not play 1 = played

^3^ McNemar-Bowker test for correlated proportions, comparing the profiles of patterns of participation in Club sport versus Other RPA. Points of greatest difference between the profiles are indicated by the use of boldface type for the higher of each pair of percentages

The first two columns of Table 1 show descriptions of the five patterns of participation, and the eight sequences of participation indicators on which the patterns are based. Then follows, for all survey respondents, profiles of counts and percentages for each pattern, for each of the three types of activity (Any RPA, Club sport, Other RPA). The McNemar-Bowker test shows significantly different profiles of participation patterns over the three years for club sport versus other RPA (*p* <.001). In Table 1 and the tables that follow, points of greatest difference between profiles are indicated by the use of boldface type for the higher of each pair or triplet of percentages. Table 1 shows that the most common pattern for participation in any form of RPA was continuous participation through 2019, 2020 and 2021. The most common pattern for participation in club sport was participation in 2019, dropout in 2020 and return in 2021 (47.8%), with very few exhibiting this pattern for participation in other RPA (3.4%). The most common pattern for participation in other RPA was continued participation throughout 2019, 2020 and 2021 (69.0%); a much smaller but nevertheless substantial proportion of club sport participants (23.5%) also exhibited this pattern. Club sport participants were also more likely to drop out in 2020 and not return in 2021 (12.0% versus 1.5%), while participants in other RPA were more likely to drop out in 2021 (10.2% versus 4.0%).

Tables 2, 3 and 4 show, for each for each of the three types of activity (Any RPA, Club sport, Other RPA), percentage profiles of patterns of participation broken down by gender, age and region of residence, together with associated chi-square tests of independence.

**Table 2:** Patterns of participation in RPA^1^ 2019–2022: by type of RPA and gender^2^

Type of RPA	Any RPA			Club sport			Other RPA		
Gender^2^	Female	Male		Female	Male		Female	Male	
Category	%	%	p-value^3^	%	%	p-value^3^	%	%	p-value^3^
Did not play in 2019	0.6	0.5	0.376	**17.4**	8.7	< 0.001	11.6	**19.4**	0.012
Dropped out 2020 no return 2021	1.6	2.6		12.0	11.9		1.6	1.5	
Dropped out 2020, returned 2021	12.0	15.0		44.2	**50.8**		3.7	3.2	
Dropped out 2021	4.8	3.9		3.3	4.7		10.3	10.2	
Continued 2019–2021	81.0	78.1		23.1	23.9		**72.9**	65.8	
N	516	620		516	620		516	620	

^1^ Recreational Physical Activity

^2^ Four gender response categories were provided: ‘Male’, ‘Female’, ‘Other’, and ‘Choose not to respond’. There were no instances of ‘Choose not to respond’, and a count of 2 for the ‘Other’ category. With such a small sample size, the evidence base is small and the sampling variability cannot be reliably estimated, and so results for this category are not included in this table

^3^ Chi-square test comparing patterns of participation between genders for each type of RPA. Points of greatest difference between the significantly different profiles are indicated by the use of boldface type for the higher of each pair of percentages

Table 2 shows that there were no significant gender differences regarding participation in any form of RPA, but there were significant gender differences regarding participation both in club sport (*p* <.001) and in other forms of RPA (*p* =.012). While the most common pattern of club sport participation was dropout in 2020 and return in 2021 for both genders, the proportion exhibiting this pattern was higher for male participants (50.8%) than female participants (44.2%). Conversely, a higher proportion of female respondents did not play club sport in 2019 (17.4% versus 8.7%). The most common pattern of participation in other RPA was continuous participation through 2019, 2020 and 2021 for both genders, but the proportion exhibiting this pattern was higher for female participants (72.9%) than male participants (65.8%). Conversely, a higher proportion of male respondents did not participate in other RPA in 2019 (19.4% versus 11.6%).

**Table 3:** Patterns of change in participation in RPA^1^ 2019–2022: by type of RPA and age group

Type of RPA	Any RPA				Club sport				Other RPA			
Age^2^ (yrs)	13–17	18–59	60+		13–17	18–59	60+		13–17	18–59	60+	
Category	%	%	%	p-value^3^	%	%	%	p-value^3^	%	%	%	p-value^3^
Did not play in 2019	0.0	0.9	0.3	< 0.001	1.1	**25.3**	4.2	< 0.001	9.9	8.6	**22.6**	< 0.001
Dropped out 2020	0.0	1.7	2.6		8.8	13.8	10.8		1.1	1.1	1.9	
Dropped out 2020, returned 2021	11.0	7.8	**19.0**		31.9	35.2	**61.0**		7.7	3.5	2.8	
Dropped out 2021	7.7	4.1	4.2		13.2	3.7	2.6		13.2	8.2	11.7	
Continued 2019–2021	81.3	**85.5**	73.9		**45.1**	22.0	21.4		68.1	**78.6**	61.0	
N	91	463	574		91	463	574		91	463	574	

^1^ Recreational Physical Activity

^2^ Ten cases had missing or incomplete dates of birth

^3^ Chi-square test comparing patterns of participation between age groups for each type of RPA. Points of greatest difference between the profiles are indicated by the use of boldface type for the highest of each triplet of percentages

Table 3 shows that there were significant age-related differences regarding participation in any form of RPA (*p* <.001), in club sport (*p* <.001) and in other forms of RPA (*p* <.001). The most common pattern of participation in any form of RPA and also in forms of RPA was continuous participation through 2019, 2020 and 2021, and the age group with the highest proportion exhibiting this pattern was 18–59 year-olds in each case (85.5% and 78.6%). The the age group with the lowest proportion was 60 + year-olds in each case (73.9% and 61.0%). For club sport, the age group with the highest proportion participating continuously through 2019, 2020 and 2021 was 13–17 year-olds (45.1%), while the age group with highest proportion dropping out in 2020 and returning in 2021 was 60 + year-olds (61.0%). Regarding 2019, the age group with the highest proportion who did not play club sport was 18–59 year-olds (25.3%), and the age group with the highest proportion who did not participate in other RPA was 18–59 year-olds (22.6%).

**Table 4:** Patterns of participation in RPA^1^ 2019–2022: by type of RPA and region^2^

Type of RPA	Any RPA			Club sport			Other RPA		
Region	Metro	Non-metro		Metro	Non-metro		Metro	Non-metro	
Category	%	%	p-value^3^	%	%	p-value^3^	%	%	p-value^3^
Did not play in 2019	0.8	0.0	0.058	13.3	11.6	0.545	14.6	18.3	0.371
Dropped out 2020 no return 2021	2.2	1.9		11.4	13.0		1.8	1.0	
Dropped out 2020, returned 2021	11.9	**16.9**		46.8	49.4		3.8	2.9	
Dropped out 2021	4.2	4.8		4.6	3.1		10.4	9.9	
Continued 2019–2021	**80.8**	76.4		23.9	22.9		69.4	68.0	
N	720	415		720	415		720	415	

^1^ Recreational Physical Activity

^2^ Regions are defined on the basis of residential postcode. The Metropolitan region comprises the Greater Capital City Statistical Area of each Australian state and territory (Ref 22 in previous paper). The Non-metropolitan region comprises the remainder of Australia. Three cases had missing postcodes

^3^ Chi-square test comparing patterns of participation between regions for each type of RPA. Points of greatest difference between the most different pair of profiles are indicated by the use of boldface type for the higher of each pair of percentages

Table 4 shows that the only substantial difference between the participation patterns of metropolitan and non-metropolitan regions was for ‘Any RPA’. A higher proportion of metropolitan respondents participated continuously through 2019, 2020 and 2021 (80.8% versus 76.4%) and a higher proportion of non-metropolitan respondents dropped out in 2020 and returned in 2021 (16.9% versus 11.9%), however the differences were not statistically significant (*p* =.058).

## Discussion

This study demonstrated that the patterns of participation in organised club-based sport and other informal sport and recreation physical activities changed significantly pre, during and post-COVID-19. There were considerable differences between the patterns of club-based sport and other recreational sport and physical activities. There is very limited research published post-COVID-19 restrictions with which to compare these results.

Most individuals who participated in recreational physical activity (69%) were able to and did participate continuously throughout the COVID-19 pandemic. However, and not surprisingly the club-sport participants were forced to dropout in 2020 during COVID-19 restrictions, and less than half reported returned to play post-COVID-19. Less than a quarter of sports club participants were able to continue to play throughout COVID-19 and beyond.

There were also nuances according to age and gender, but not so much difference in the participation patterns according to residential location. More males (51%) returned to playing sport post-COVID compared to females (44%) which is consistent with other Australian data [9]. The reasons for this are unclear. We know that families finances have been negatively impacted during COVID-19 contributing to financial burden to returning to play sport [24], however this should not have a gendered lens to it. We propose a few possible explanations for lower return to playing sport for females compared to males. Given the complexity of sport behaviour determinants [25, 26], it is likely that there are multiple reasons and influences that are impacting females more than males when it comes to returning to play sport.

The sport and physical activity profiles do differ for boys and girls, and boys are generally more likely to play competitive club-based sport [27, 28], with participation in club-based sport playing a smaller role in terms of total physical activity as females approach adolescence [29]. Further, girls generally perceive themselves as less competent compared to boys [29], and in general lower participation in sport for girls can be indicative of less positive family support [29]. Perhaps females were less motivated and driven to return to playing club-based sport post-COVID-19 and they may lack the confidence, competence and family support to return to play compared to males. Further, during adolescence females are more likely to drop out compared to males [30, 31], and perhaps COVID-19 has just exacerbated this phenomenon with females are dropping out earlier.

Another contributing factor to the gender differences in returning to play club-based sport may be the health of individuals. The inaccessibility of community sport and schools and home confinement during COVID-19 restrictions deprived children and youth of physical activity, and created experiences of isolation, loneliness and grief [32]. However, amongst sports participants, female youth in Australia were reportedly more impacted by COVID-19 with poorer physical, general and mental health compared to males [16].

For club sport, the age group with the highest proportion participating continuously through 2019, 2020 and 2021 was 13–17 year-olds (45.1%). This is a favourable finding because historically we have always seen considerable dropout of sport during late adolescence [30, 33, 34]. The age group with the highest dropout in 2020 and return in 2021 was the older adults, aged 60+, with over 60% returning to play. Older adults are more likely to have more leisure-time available perhaps less financial stress than working adults with young families, and this may have contributed to higher return rates [35].

Participation in community club-based sport can provide significant positive social and mental health and wellbeing above and beyond physical health benefits and individual-based activities. This is largely due to the social nature of team and club-based sport. As Michelini et al., (2023) recently questioned, “Will society (and if so how) commit to processes aimed at generating and rewarding collaborative behaviour and not only competitive and speculative ones?” Driving community engagement through participation in sport has the ability to provide a wealth of positive personal development as well as a range of health and wellbeing outcomes.

Priority and specific attention should be given to understanding why Australian women and girls have not returned to playing community club-based sport to the same extent that Australian boys and men have. Further, as our society’s preferences for engagement in sport and physical activity are evolving, we need to ensure that opportunities to play sport match individuals preferences to play. The Sport4Me conceptual model proposes a new delivery model of community sport which focuses on the key motivations of individuals including friends, fun, physical literacy, flexibility and play [36].

### Limitations

In any survey-based study, the extent to which the survey sample is representative of the population from which the sample is drawn, or of related populations with different contextual characteristics, is an issue which must be considered when interpreting the study findings. In the present study, the risk of selection bias, including self-selection or response bias, occurred at two stages. The first survey sample was a convenience sample, predominantly of Australian sports participants recruited with the assistance of Australian national and state sporting organisations (NSOs and SSOs) of several popular sports, supplemented by recruitment through social media, which resulted in an additional smaller sample of participants in only informal sport or other physical activity settings [22]. Recruitment for the first survey was thus multi-facetted, and mostly facilitated by third parties, making it impossible to ascertain a response rate. The follow-up survey sample was a self-selected subset of the first sample. Recruitment for the follow-up survey was conducted directly by the research team, with a response rate of 21.2%. Given these limitations, caution should be exercised in generalising the results of the study. Notwithstanding that, on the other side of the ledger, the sample obtained was large, and because respondents provided information about the multiple sports and other physical activities that they engaged in, there was comprehensive representation of the sporting codes and other types of recreational physical activity that are available in Australia.

## Conclusion

The sport and physical activity behaviours of individuals were severely impacted by COVID-19 restrictions. This study has demonstrated that many Australians were able to continue to be active through non-sporting, individual-based activities. However, participation in community club-based sport has been significantly negatively impacted by COVID-19, more so than participation in some other recreational sport and physical activities. Further, fewer females than males returned to playing community sport, and priority and specific attention should be given to understanding why women and girls have been less likely to return to playing community club-based sport.

## Data Availability

The datasets used and/or analysed during the current study are available from the corresponding author on reasonable request.

## Abbreviations

RPA: Recreational Physical Activity
